# A Left-sided Azygos Vein in a Cadaver: Anatomical and Surgical Considerations

**DOI:** 10.7759/cureus.2610

**Published:** 2018-05-10

**Authors:** Konstantinos Koutsouflianiotis, George K Paraskevas, Kalliopi Iliou, George Noussios

**Affiliations:** 1 Department of Anatomy and Surgical Anatomy, Aristotle University of Thessaloniki

**Keywords:** azygos vein, left side, anatomy, variation

## Abstract

Despite the wide-spread knowledge among anatomists and surgeons that the azygos vein lies on the right side of the vertebral column, various scientific works have been conducted which suggest the existence of left-sided azygos veins. The displacement of the vessel seems to be related with aging, due to crossover veins and the development of osteophytes on the thoracic vertebrae. The current case report confirms the variation of the azygos vein’s course, highlights the awareness of the relatively unusual left-sided location of the azygos vein for the surgeon of the region, and underlines the clinical significance of such knowledge to the modern internist-radiologist, general surgeon and thoracic surgeon, as well.

## Introduction

It is widely known to the anatomists and surgeons that the azygos vein (AV) lies on the right side of the vertebral column. Many classic surgical anatomical textbooks describe that the AV normally ascends on the right side of the vertebral column, arching anteriorly and medially before it drains into the superior vena cava [1-5]. The abovementioned report is under dispute since scientific works are published which indicate the existence of the AV on the left side of the vertebral column, mainly in older adults.

Our case report describes a relatively enlarged AV on the left side of the spine from tenth to eighth thoracic vertebra confirming the variability in the course of the vessel. The morphological characteristics of our variant, its embryological origin, its clinical applications, and the relevant literature are discussed as well.

## Case presentation

**Figure 1:**
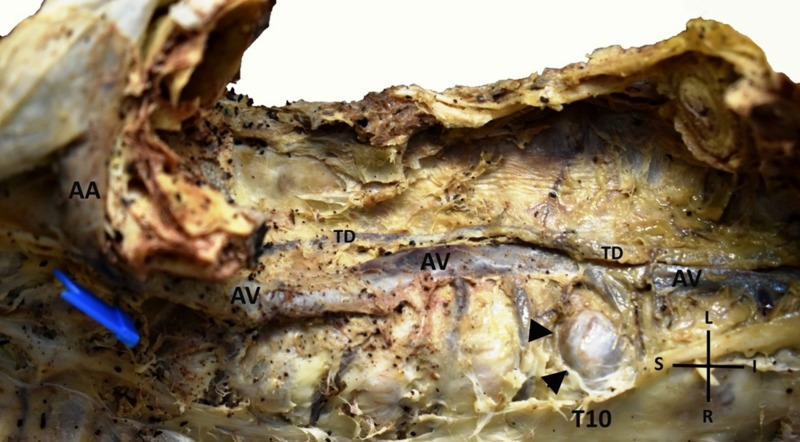
An enlarged azygos vein (AV) is demonstrated coursing on the left aspect of the vertebral column (right- superior view) (AV: azygos vein, TD: thoracic duct, AA: azygos arch, T10: tenth thoracic vertebra, arrow heads: osteophyte, S: superior, I: inferior, R: right, L: left)

**Figure 2:**
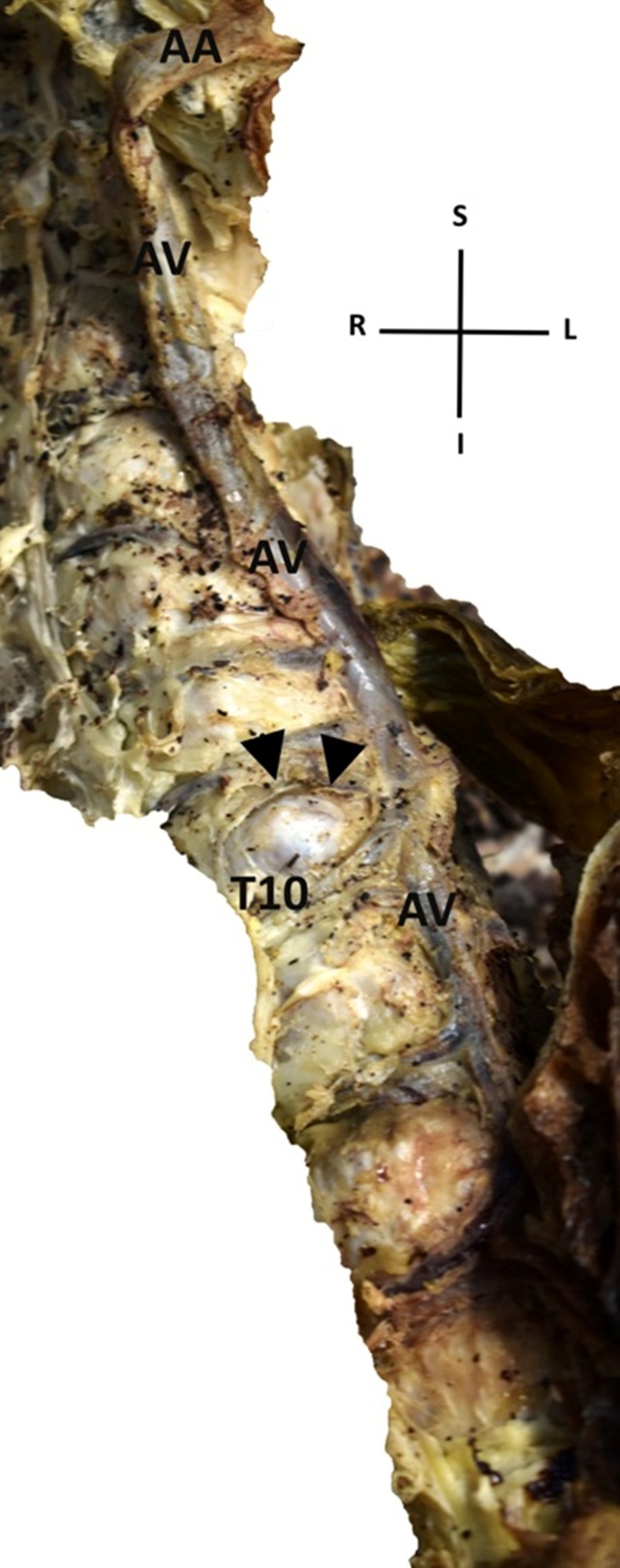
An enlarged azygos vein (AV) is demonstrated coursing on the left aspect of the vertebral column (Right-inferior view of the abovementioned AV’s variant) (AV: azygos vein, AA: azygos arch, T10: tenth thoracic vertebra, arrow heads: osteophyte, S: superior, I: inferior, R: right, L: left)

During a routine dissection in our Department of Anatomy and Surgical Anatomy, we encountered the presence of the AV on the left side of the vertebral column (Figure 1 and Figure 2].

The dissection was conducted on an 80-year-old formalin-fixed female cadaver, used for educational and research purposes, whose death was unrelated to the present case report. Specifically, after the meticulous dissection of the thorax region and the mediastinum, and after the excision of both lungs and the heart by means of the classical method of anatomical dissection, we detected an enlarged left-sided AV with maximum diameter 11.05 mm approximately at its midportion. The specific cadaver displayed hemiazygos and accessory hemiazygos veins in the expected course in the mediastinum, whereas the thoracic duct was noted ascending normally through the posterior mediastinum between the azygos vein and the thoracic aorta. In particular, at the level of tenth, ninth, and eighth thoracic vertebra the AV lay on the left side of the spine and the distance between the vessel and the midline of the vertebral column was 3.91 mm at the tenth, 5.75 mm at the ninth and 2.69 mm at the eighth thoracic vertebra. From the twelfth to tenth thoracic vertebra the AV lay on the midline of the spine, whilst from the seventh thoracic vertebra to its end in the superior vena cava, the AV was found as expected at the right side of the vertebral column. Our finding was documented by several photographs taken using a Nikon D3400 digital camera, and the measurements were made using a digital vernier caliper with an accuracy of 0.01 mm. No other congenital anomalies, variations or pathological conditions, or evidence of previous surgical interventions in the region were present.

## Discussion

The origin of the AV is not constant. It is often formed by the union of the right ascending lumbar vein and the right subcostal vein. It penetrates the diaphragm through the aortic opening [2] and it ascends in the posterior mediastinum, passing close to the right side of the bodies of the inferior eight thoracic vertebrae, arching over the superior aspect of the root of the right lung to join the superior vena cava [3]. On the other hand, the hemiazygos vein inferiorly and the accessory hemiazygos vein superiorly, lie longitudinally on the left side of the bodies of the thoracic vertebrae [5]. From embryological aspect, both AV and hemiazygos veins originate from supracardinals veins, which become broken in the region of the kidneys at the eighth week of development, and then they unite by a cross anastomosis to become AV and hemiazygos vein [6].

We consider that our case report regarding a left-sided AV is of a great anatomical and clinical significance since, as it is already mentioned, most anatomical textbooks describe the course of the ascending AV being normally on the right aspect of the vertebral column, on the right side of the aorta and anterior to the right intercostal arteries. Nathan studied the course of the AV in 150 dissected cadavers and came to the conclusion that in stillborn infants the percentage of a left-sided AV is low (15%, 3 in 20 examined infants). On the contrary, in adult cadavers, a percentage of 53% (69 in 120 cadavers) presented an AV crossing to the left [7]. To the same conclusion came other researchers who studied the course of the AV in adults and infants. Specifically, Sarnowska et al. did not find any AV on the left side of the vertebral column in stillborn fetuses [8], while Rokutanda et al. came across a left-sided AV only in 2%, in a sample of 50 six-month-old infants [9].

Apart from Nathan, Saito et al. with a sample of 47 adult cadavers (mean age 84.7 years old), found the AV on the left in 44 cadavers (94%). Tokutome, on the other hand, after dissecting a number of 54 young adult cadavers (mean age 42 years old) came across only 4% of left-sided AV [9]. Tatar et al. examined the course of the AV in chest CT's of 103 cases, with a mean age 47.08 years old, and found the AV on the right side of the body in 39 cases (37.9%); in 23 cases (22.3%), the AV was on the left side of the vertebral column; and in the remaining 41 cases (39.8%), the AV was located at the midline and in front of the vertebral column [10]. The above results state that in young ages (infants and young adults) the course of the AV is as expected on the right or the middle of the vertebral column, whereas in elderly one can find the vessel displaced on the left of the spine. Thus, it can be proposed that aging could cause such displacements [9].

Possible explanations for the anomalous course of the AV have to be given, beyond the rare occasion of independent left AV (persistence of the early embryonic form) [11]. Bales attributed the displacement of the AV to crossover veins, which gradually over time transpose the vessel from its regular course [12]. Also, Nathan suggested that the existence of asymmetric osteophytes on the thoracic vertebrae could explain the deviation of the AV due to mechanical pressure to the left [7]. In our case report, an osteophyte is developed on the tenth thoracic vertebra, presumably dislocating the AV to the left side of the vertebral column.

## Conclusions

The knowledge of such variations is an important asset to the modern internist-radiologist who face the challenge of differential diagnosis between mediastinal tumors and other lung pathologies and the surgeon of the thorax as well in order to avoid implications such as hemorrhage when a left-sided AV exists.
